# The College reply to Francis misses the big question: a commentary on OP92

**DOI:** 10.1192/pb.bp.114.047514

**Published:** 2014-08

**Authors:** John Cox, Alison Gray

**Affiliations:** 1 Keele University, UK; 2 2gether NHS Foundation Trust, Hereford, UK

## Abstract

The College has recently published an occasional paper in response to the Francis inquiry into the care at Mid Staffordshire NHS Foundation Trust. We consider that it overlooks one key question implicit in the inquiry’s recommendations: ‘Is the business model of care fit for purpose?’ We question whether the business model in its present form is appropriate for the delivery of healthcare. We suggest there is a need for greater conceptual clarity with regard to the nature of compassionate care and the meaning of person-centred medicine. We recommend that a broader moral and ethical framework is considered not only for psychiatry, but for all healthcare provision which would transcend specialty and Royal College boundaries.

In the aftermath of the Francis inquiry into the failure of care at the Mid Staffordshire NHS Foundation Trust, profound political and existential questions are raised. Has the National Health Service (NHS) lost its moral compass? Has the price paid for general management been too high? Is a relationship-based medicine of the person impossible in a postmodern, industrialised healthcare system?

## A missed opportunity

The Francis report, with its catalogue of total system failure, was profoundly shocking - and especially so for the families of those who died unnecessarily or whose basic care was neglected; up to 1200 excess patient deaths have been reported.^1^ The report called for compassionate care throughout the NHS, but did not explain how such compassion was to be cultivated or measured. The response from the Royal College of Psychiatrists to the Francis recommendations - occasional paper OP92^2^ - was not, however, a radical call for change in NHS structure, nor did it consider the question implicit in the Francis report: ‘Is the business model for healthcare fit for purpose?’ Yet Sue Bailey, in her robust foreword as College President, was strident in her indictment of the Trust’s total failure to put patients first, and pointed to the danger of apathy and learned helplessness among psychiatrists. The College’s response, however, indirectly highlighted the lack of conceptual thought about what constitutes compassionate empathy and care, and what are the philosophical, religious and existential understandings of personhood and person-centred medicine. The extent to which practitioners’ compassionate values go deeply enough, and are derived from healthy moral communities, are other questions that could have been addressed.

## Wide-ranging evidence of failure

The College’s Centre for Quality Improvement (CCQ I) is tasked with considering many of these key questions - and their work could be foundational. Many faith groups have traditions and current practices that are useful resources for compassion,^3^ and the College in its report has correctly acknowledged the need for professionals’ well-being to be considered alongside that of the patient. In our experience it is challenging and stressful to maintain compassion in the face of overwhelming workloads, shortage of staff, an inappropriate team mix and intrusive management. These factors each need to also be addressed.

The proposal, laid out in OP92, to publish a code of ethics for College members is welcome, but this should also provide a broad moral and ethical framework for all healthcare. For example, Gilbert *et al*^4^ question the moral integrity of the NHS on the grounds of lack of meaningful patient choice and limited evidence for efficiency savings. We, too, describe the erosion of professional idealism and the effect of the target-driven mindset that can lead to the loss of vocation and of compassion.^5^ We, too, question whether the business model in its present form is fit for purpose; community psychiatry - historically, the bastion of imaginative innovation in mental healthcare - has become ossified by the shift of control from clinician to management.^6^ Other research suggests that lack of control causes ‘flame out’ by lowering self-directedness and reducing self-transcendence.^7^ There is dissonance between the business and medical models of care, which can precipitate poor care for the patient, and work failure, family breakdown and personal tragedy for the clinician.^8,9^

## The way forward

The College plans to update its response to the Francis inquiry in 6 months. Collaboration in this effort with other medical Royal Colleges and with other professional and patient groups, as well as with moral philosophers, is highly desirable. There is much imaginative writing in this field, not only from moral philosophers but also from ethicists and comparative religion experts,^3,10^ which will help to sharpen the conceptual understanding of patient-centred care. If this collaboration is outside the remit of the Academy of Medical Royal Colleges, then it could be outsourced to an alliance of other healthcare-related organisations, similar to the Mental Health Alliance (www.mentalhealthalliance.org.uk).

David Owen^11^ has called for emergency legislation to be enacted in 2015, within 3 months of the general election, to reinstate the NHS as provider of a comprehensive service and to get rid of marketisation of healthcare. The College should join this national debate, and consider Chronos and Kairos when updating OP92. Chronos demands the update to be completed within 6 months, which could be facilitated by a survey of College members’ opinion. Yet this is also a crucial Kairos moment - a pivotal flashpoint for medicine as a whole - as it considers whether the business model itself is fit for purpose. It is an opportunity not to be missed.
